# A Composite Histopathologic Invasion Score Predicts Lymph Node Metastasis in Gastric Cancer

**DOI:** 10.3390/jcm15176509

**Published:** 2026-08-22

**Authors:** Ebru Karcı, Mahya Ahmadpour Youshanlui, Ferhat Ozden, Ozgur Acikgoz, Ömer Fatih Ölmez, Ozcan Yildiz, Hafize Uzun, Ahmet Bilici

**Affiliations:** 1 Department of Medical Oncology, Faculty of Medicine, Istanbul Atlas University, 34408 Istanbul, Türkiye; 2 Department of Medical Biochemistry, Faculty of Medicine, Istanbul Atlas University, 34408 Istanbul, Türkiye; mahyaahmadpouryoushanlui@gmail.com (M.A.Y.); hafize.uzun@atlas.edu.tr (H.U.); 3 Department of Pathology, Faculty of Medicine, İstanbul Medipol University, 34403 Istanbul, Türkiye; ferhat.ozden@medipol.edu.tr; 4 Department of Medical Oncology, Faculty of Medicine, İstanbul Medipol University, 34403 Istanbul, Türkiye; ozgur.acikgoz@medipol.edu.tr (O.A.); abilici@medipol.edu.tr (A.B.); 5 Department of Medical Oncology, Faculty of Medicine, İstanbul Acibadem Mehmet Ali Aydinlar University, 34638 Istanbul, Türkiye; ofolmez@medipol.edu.tr; 6 Department of Medical Oncology, Faculty of Medicine, Biruni University, 34015 Istanbul, Türkiye; oyildiz@medipol.edu.tr

**Keywords:** gastric adenocarcinoma, lymph node metastasis, lymphovascular invasion, perineural invasion, extracellular matrix remodeling, *MMP11*, *FAP*

## Abstract

**Background:** Lymph node metastasis (LNM) is a major determinant of prognosis in gastric adenocarcinoma. Although lymphovascular invasion (LVI) and perineural invasion (PNI) are established markers of aggressive tumor behavior, their combined association with nodal dissemination has not been systematically evaluated. This study investigated the relationship between invasive histopathologic features and LNM and developed a composite invasion score. **Methods:** This retrospective study included 395 patients with gastric adenocarcinoma. Clinicopathological analyses involving LNM were performed in the surgical resection cohort. A composite invasion score (0–2) was generated based on the presence of LVI and PNI. Associations with LNM, metastatic lymph node burden, and lymph node ratio (LNR) were evaluated using non-parametric tests and multivariable logistic regression. Predictive performance was assessed using receiver operating characteristic (ROC) analysis. An exploratory external transcriptomic analysis was performed using the GSE15459 dataset. **Results:** Both LVI and PNI were significantly associated with LNM (both *p* < 0.001). The composite invasion score showed a stepwise association with nodal dissemination, with median metastatic lymph node counts increasing from 0 in score-0 tumors to 3.5 in score-1 and 9 in score-2 tumors (*p* < 0.001). After adjustment for pathological T stage, histological grade, Lauren classification, age, and tumor location, the composite invasion score remained an independent predictor of LNM (OR = 4.53, 95% CI: 1.81–13.00, *p* = 0.002). LNR also increased significantly across score categories (*p* < 0.001). The composite invasion score demonstrated good discriminatory performance for LNM (AUC = 0.848, 95% CI: 0.773–0.923). The exploratory transcriptomic analysis demonstrated significant upregulation of *MMP11*, *MMP14*, and *FAP* in advanced-stage tumors, consistent with increased extracellular matrix remodeling and stromal activation. **Conclusions:** A simple composite invasion score integrating LVI and PNI is independently associated with lymph node metastasis and metastatic burden in gastric adenocarcinoma. The accompanying exploratory transcriptomic analysis provides biological context for these clinicopathological findings by demonstrating stage-associated extracellular matrix remodeling rather than direct molecular validation of the proposed score. These findings support the potential utility of the composite invasion score as a postoperative pathology-derived risk stratification tool.

## 1. Introduction

Gastric cancer remains a major global health burden and is among the leading causes of cancer-related mortality worldwide [1]. Prognosis is largely determined by local tumor invasion and metastatic dissemination, particularly lymph node metastasis, which remains a cornerstone of staging, treatment selection, and survival prediction. Nevertheless, patients with similar TNM stages may exhibit markedly different clinical outcomes, suggesting that anatomic staging alone may not fully reflect tumor aggressiveness.

Tumor invasion and nodal dissemination represent sequential steps of the metastatic cascade. Among routine histopathologic features, lymphovascular invasion (LVI) and perineural invasion (PNI) are established markers of aggressive tumor behavior and have been associated with lymph node metastasis and adverse outcomes in gastric cancer [2,3]. Although molecular biomarkers such as HER2, PD-L1, and mismatch repair (MMR) status are increasingly used for therapeutic stratification, their value in predicting nodal dissemination is limited compared with direct indicators of tumor invasion [4].

At the molecular level, metastatic progression is closely linked to extracellular matrix (ECM) remodeling and stromal activation. ECM remodeling promotes cancer cell migration, angiogenesis, lymphangiogenesis, and metastatic spread [5]. In gastric cancer, ECM-related phenotypes have been associated with poor prognosis and epithelial–mesenchymal transition (EMT) features [6]. Matrix metalloproteinases, particularly *MMP11* and *MMP14*, contribute to ECM degradation and tumor invasion, whereas fibroblast activation protein alpha (*FAP*) plays a central role in cancer-associated fibroblast activation and tumor progression [7,8,9,10].

Despite growing evidence supporting the roles of invasive histopathologic and molecular features, their integrated relationship with nodal metastatic burden remains insufficiently understood. Therefore, this study investigated clinicopathologic and transcriptomic factors associated with lymph node metastasis in gastric adenocarcinoma. We specifically evaluated whether a composite invasion score based on routine histopathologic findings could serve as a practical tool for nodal risk stratification and whether its biological basis could be supported by transcriptomic evidence of ECM remodeling and stromal activation.

## 2. Materials and Methods

### 2.1. Study Design and Patient Cohort

This retrospective study included patients diagnosed with gastric adenocarcinoma who were evaluated at Medipol Mega University Hospital between 2015 and 2022. A total of 395 cases were included based on the availability of clinicopathologic and histopathologic data.

Patient demographic characteristics, including age and sex, were recorded. Pathologic data were retrieved from institutional records and included specimen type (biopsy or resection), tumor location, histologic subtype according to Lauren classification (intestinal, diffuse, or mixed), tumor grade, depth of invasion (pT stage), lymph node status, and the presence of LVI and PNI. Clinicopathological analyses involving lymph node metastasis, metastatic lymph node burden, lymph node ratio (LNR), and the composite invasion score were restricted to patients who underwent surgical resection. Analyses of the composite invasion score included only cases with available assessments for both LVI and PNI.

### 2.2. Histopathologic Evaluation and Biomarker Assessment

Histopathologic evaluation was performed using hematoxylin and eosin (H&E)-stained sections according to standard diagnostic criteria. LVI was defined as the presence of tumor cells within endothelial-lined vascular or lymphatic spaces, while PNI was defined as tumor cell infiltration within or surrounding nerve fibers.

Biomarker data, including HER2, PD-L1, mismatch repair (MMR) protein status, and Helicobacter pylori status, were obtained from pathology reports where available. HER2 status was assessed by immunohistochemistry and/or in situ hybridization. PD-L1 expression was evaluated using immunohistochemistry and interpreted based on the combined positive score (CPS). MMR status was determined by immunohistochemical expression of MLH1, PMS2, MSH2, and MSH6 proteins, with intact expression classified as mismatch repair proficient (pMMR/MSS). H. pylori status was determined by histologic or ancillary testing methods. Because biomarker testing was performed according to clinical indications, not all biomarkers were available for every patient.

### 2.3. Definition of Nodal Parameters

Lymph node metastasis was defined as the presence of tumor involvement in at least one regional lymph node. The total number of examined lymph nodes and the number of metastatic lymph nodes were recorded for resection specimens.

The lymph node ratio (LNR) was calculated as the ratio of metastatic lymph nodes to the total number of examined lymph nodes. Cases with zero examined lymph nodes were excluded from LNR-related analyses.

### 2.4. Composite Invasion Score

To integrate key histopathologic indicators of tumor invasiveness, a composite invasion score was constructed based on the presence of LVI and PNI. Each feature was assigned a value of 1 if present and 0 if absent, resulting in a cumulative score ranging from 0 to 2. Tumors were categorized into three groups (score 0, 1, or 2) to evaluate stepwise associations with nodal metastasis and metastatic burden. Because the score was derived exclusively from postoperative histopathological findings, it was evaluated as a postoperative pathological risk stratification tool and was not intended for preoperative prediction of lymph node metastasis.

### 2.5. Bioinformatic Analysis

To provide external molecular context for the clinicopathological findings, an exploratory external transcriptomic analysis was performed using the publicly available Gene Expression Omnibus (GEO) dataset GSE15459, which includes gene expression profiles of primary gastric adenocarcinoma samples. Samples were stratified into early-stage (stage I–II, *n* = 59) and advanced-stage (stage III–IV, *n* = 133) groups based on available clinical staging information. Differential gene expression analysis between these two groups was conducted using the GEO2R online analysis tool. Genes of interest were selected based on their established roles in extracellular matrix (ECM) remodeling, tumor invasion, epithelial–mesenchymal transition (EMT), and hypoxia-related pathways. The Benjamini–Hochberg method was applied to control for the false discovery rate, and adjusted *p* values (adj.P.Val) were reported. Genes with adj.P.Val < 0.05 were considered statistically significant. Because GSE15459 does not include annotations for lymphovascular invasion (LVI), perineural invasion (PNI), lymph node metastasis, or the composite invasion score, this analysis was designed to characterize stage-associated ECM remodeling rather than to directly validate the proposed pathological scoring system. The full list of differentially expressed genes obtained from the GEO2R analysis is available in Supplementary File S1.

### 2.6. Ethical Considerations

This retrospective study was conducted in accordance with the principles of the Declaration of Helsinki. The study protocol was approved by the Istanbul Atlas University Non-Interventional Scientific Research Ethics Committee (approval date: 11 May 2026; approval number: E-22686390-200-99863). The requirement for informed consent was waived due to the retrospective nature of the study. Clinical and pathological data were retrieved from a prospectively maintained institutional database. All eligible patients diagnosed between January 2015 and November 2022 were consecutively identified to minimize selection bias.

### 2.7. Statistical Analysis

All statistical analyses were performed using R version 4.5.3 (R Foundation for Statistical Computing, Vienna, Austria). Categorical variables were summarized as counts and percentages, whereas continuous variables were reported as mean ± standard deviation (SD) or median with interquartile range (IQR), as appropriate. For comparisons of categorical variables, Fisher’s exact test was used. Continuous variables with non-normal distributions, including metastatic lymph node count and LNR, were compared across groups using the Kruskal–Wallis test. When comparisons involved two groups, the Wilcoxon rank-sum test was applied. LNR was calculated as the number of metastatic lymph nodes divided by the total number of examined lymph nodes. Cases with zero examined lymph nodes were excluded from LNR analysis. Descriptive analyses were performed using all available data for each variable; therefore, denominators varied according to data availability.

To assess independent predictors of lymph node metastasis, a multivariable logistic regression model was constructed including the composite invasion score, pathological T stage, histological grade, Lauren classification, and age. Age was treated as a continuous variable. Tumor location was not included in the multivariable model because the original variable comprised numerous anatomical categories, many of which were represented by only one or two patients, resulting in sparse data and unstable parameter estimation. Cases with missing data for variables included in the model were excluded using complete-case analysis. Odds ratios with 95% confidence intervals were reported.

To evaluate stepwise relationships across ordered groups, including composite invasion score categories, non-parametric tests were applied as described above.

The discriminatory performance of the composite invasion score for predicting lymph node metastasis was assessed using receiver operating characteristic (ROC) analysis in the same complete-case–cohort used for the multivariable logistic regression analysis. The area under the curve (AUC), 95% confidence interval (CI), optimal cutoff value, sensitivity, and specificity were calculated. ROC analyses were also performed for pathological T stage, lymphovascular invasion (LVI), and perineural invasion (PNI), and pairwise comparisons between ROC curves were conducted using DeLong’s test.

Analyses involving lymph node metastasis, metastatic lymph node burden, LNR, and the composite invasion score were restricted to patients who underwent surgical resection.

All tests were two-sided, and a *p* value of <0.05 was considered statistically significant.

## 3. Results

### 3.1. Cohort Characteristics and Biomarker Landscape

The overall cohort comprised 395 patients (Table 1). The mean age was 66.0 ± 13.9 years, with a median age of 67 years (IQR, 57–76). Most patients were male (283/395, 71.6%), while females accounted for 112/395 cases (28.4%). Biopsy specimens constituted the majority of samples (268/395, 67.8%), whereas 127/395 specimens (32.2%) were surgical resection specimens. Among tumors with available Lauren classification, the intestinal subtype was the most frequent (*n* = 73), followed by diffuse (*n* = 52) and mixed (*n* = 11) histology.

Biomarker profiling demonstrated *H. pylori* positivity in 55/149 tested cases (36.9%), PD-L1 positivity in 25/61 tested cases (41.0%), and HER2 positivity in 47/395 cases (11.9%). Mismatch repair analysis showed that 38/43 tested tumors (88.4%) were MSS/pMMR, indicating preserved mismatch repair protein expression in most evaluated samples.

Of the 127 surgical resection specimens, one case was excluded from the clinicopathological analyses because the required pathological information was unavailable, resulting in a final resection cohort of 126 patients (Table 2). Because some clinicopathological variables were unavailable for all patients, the denominators vary across variables and are reported for each analysis accordingly. Clinicopathological characteristics of the surgical resection cohort are summarized in Table 2. Among the resection cohort, lymph node metastasis was identified in 85 of 120 evaluable patients (70.8%). The median numbers of examined and metastatic lymph nodes were 34 (IQR, 22–45) and 4 (IQR, 0–9), respectively. Lymphovascular invasion and perineural invasion were present in 80/112 (71.4%) and 68/112 (60.7%) evaluable cases, respectively.

**Figure 1:**
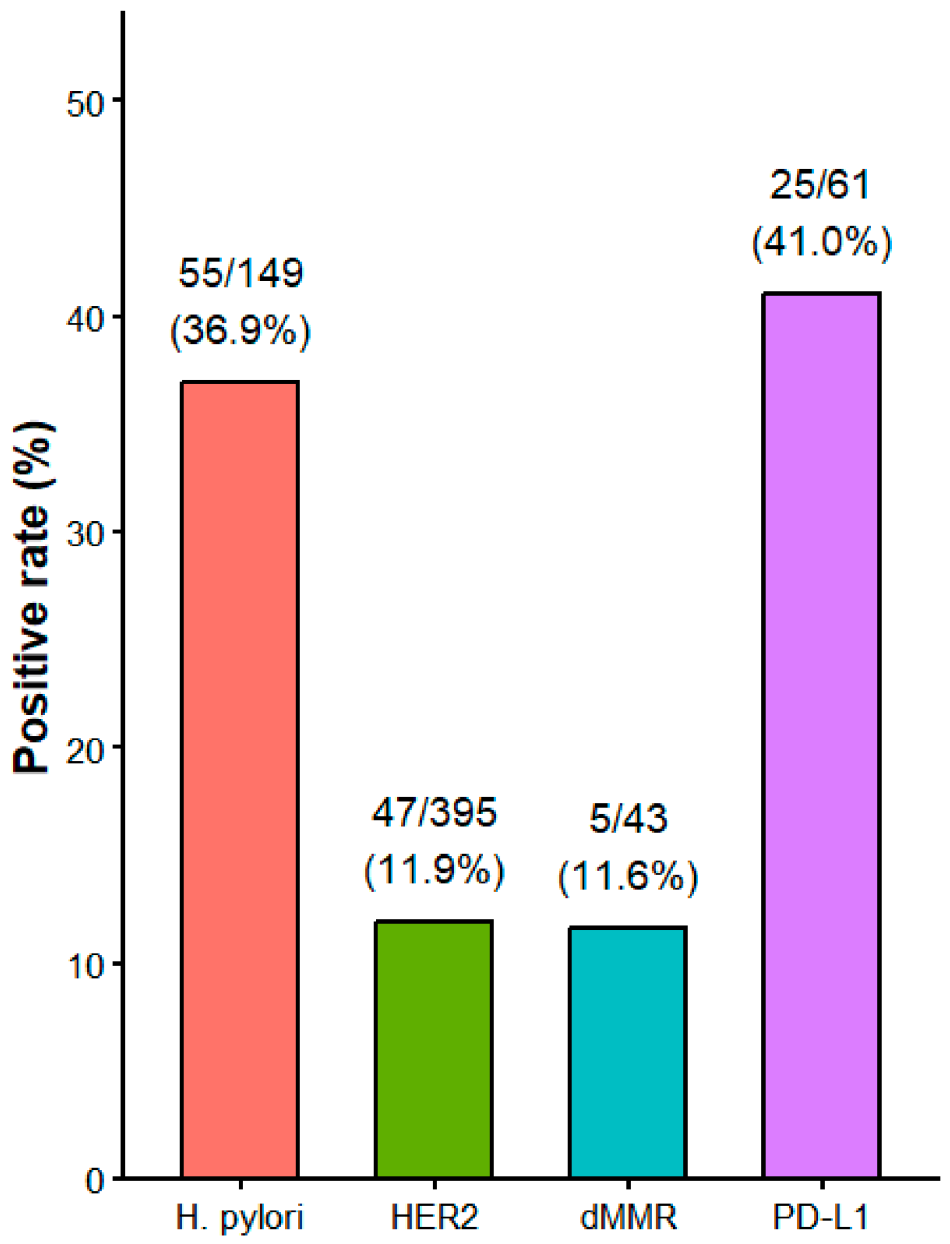
Positive rates of *Helicobacter pylori*, HER2, PD-L1, and mismatch repair deficiency (dMMR) in the study cohort. Values above each bar indicate the number of positive cases relative to the total number of patients tested for each biomarker, followed by the corresponding percentage. Because biomarker testing was performed in different patient subsets according to clinical indications, the denominators differ across biomarkers; therefore, the reported positive rates should not be interpreted as direct comparisons between biomarkers.

The distribution of biomarker positivity rates according to the number of tested cases is presented in Figure 1.

### 3.2. Conventional Clinicopathologic Correlates of Nodal Spread

Among resection specimens, both LVI and PNI demonstrated strong associations with lymph node metastasis. Tumors exhibiting LVI were significantly more likely to show nodal involvement compared with LVI-negative tumors (Fisher’s exact test, *p* < 0.001). A similarly significant relationship was observed for PNI, further supporting the role of invasive tumor behavior in facilitating regional metastatic spread (Fisher’s exact test, *p* < 0.001).

Beyond binary nodal positivity, both invasive pathologic features were associated with markedly increased metastatic lymph node burden (Table 3). Tumors without LVI showed a median of 0 positive lymph nodes, compared with 6 in LVI-positive tumors (*p* < 0.001). Likewise, tumors without PNI demonstrated a median of 1 positive lymph node, whereas PNI-positive tumors showed a median of 7 positive lymph nodes (*p* < 0.001).

**Figure 2:**
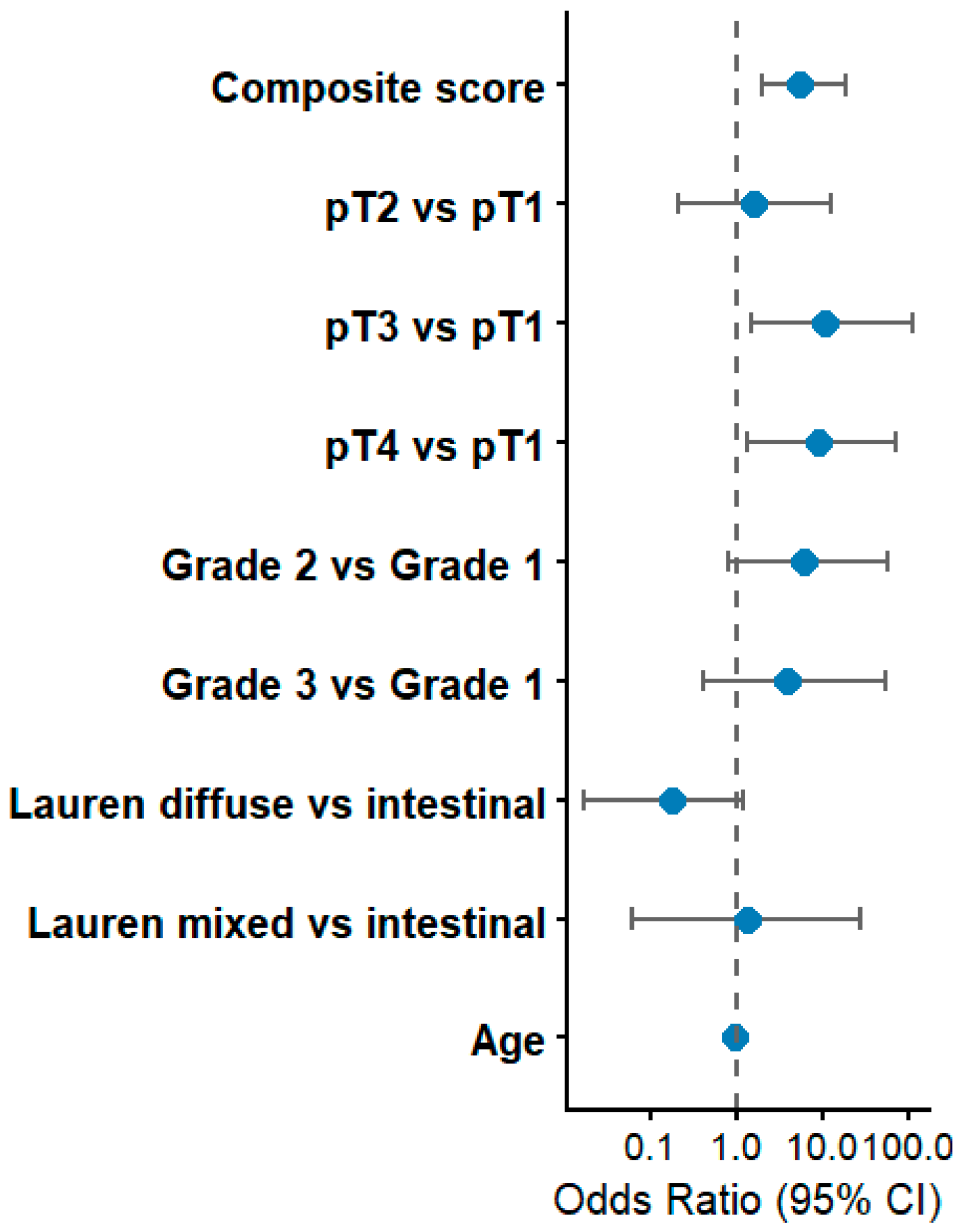
Multivariable logistic regression analysis of clinicopathological factors associated with lymph node metastasis. Forest plot showing the adjusted odds ratios and 95% confidence intervals derived from the multivariable logistic regression model. The final model included 105 patients with complete data and incorporated the composite invasion score, pathological T stage (pT), histological grade, Lauren classification, and age. The composite invasion score remained an independent predictor of lymph node metastasis after adjustment for conventional clinicopathological factors (OR = 4.53, 95% CI: 1.81–13.00, *p* = 0.002), while pathological T stage also remained independently associated with lymph node metastasis (OR = 2.10, 95% CI: 1.14–4.08, *p* = 0.020). Points represent adjusted ORs, and horizontal bars indicate 95% CIs. The dashed vertical line indicates an OR of 1.0 (no association).

To evaluate whether the composite invasion score remained independently associated with lymph node metastasis after adjustment for established clinicopathological factors, a multivariable logistic regression model was constructed using the 105 patients with complete data for all variables included in the model. The model included the composite invasion score, pathological T stage, histological grade, Lauren classification, and age (Figure 2). The composite invasion score remained an independent predictor of lymph node metastasis (OR = 4.53, 95% CI: 1.81–13.00, *p* = 0.002), while pathological T stage was also independently associated with nodal metastasis (OR = 2.10, 95% CI: 1.14–4.08, *p* = 0.020). Histological grade, Lauren classification, and age were not independently associated with lymph node metastasis.

### 3.3. Comparative Analyses Across Histologic, Treatment, and Biomarker Subgroups

Comparative analyses across Lauren histologic subtypes did not demonstrate statistically significant differences in lymph node positivity. Likewise, exploratory analyses of the available biomarker subsets, including *Helicobacter pylori* status, HER2 expression, and PD-L1 status, did not identify statistically significant associations with lymph node positivity (Table 4).

### 3.4. Metastatic Cascade Model

A clear metastatic cascade pattern emerged across local invasion depth, composite invasive phenotype, and nodal dissemination metrics. Increasing pT stage was significantly associated with greater metastatic lymph node burden (Kruskal–Wallis, *p* < 0.001) and was also strongly associated with higher composite invasion scores (Fisher’s exact test, *p* < 0.001), indicating that deeper local tumor extension parallels the accumulation of invasive histopathological features.

**Figure 3:**
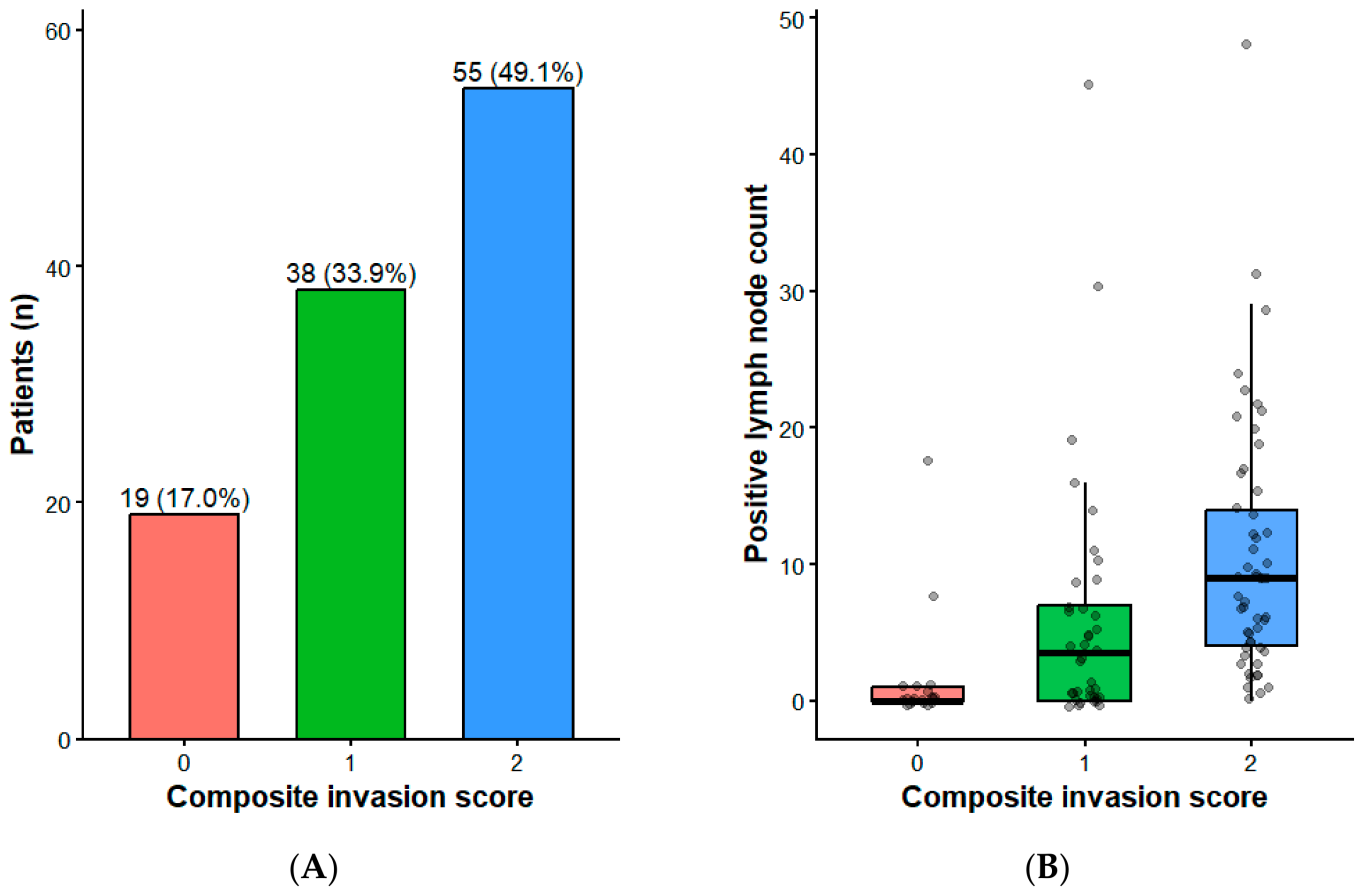
Distribution and clinical significance of the composite invasion score. (**A**) Distribution of patients according to the composite invasion score. Bars represent the number of patients in each score category, with percentages indicated above each bar. Red, green, and blue represent composite invasion scores of 0, 1, and 2, respectively; the same color coding is used in panel (**B**). (**B**) Distribution of positive lymph node counts according to the composite invasion score. Box plots show the median, interquartile range (IQR), and range excluding outliers, while individual points represent individual patients. Increasing composite invasion scores were associated with progressively higher positive lymph node counts, indicating a greater metastatic burden.

The composite invasion score demonstrated a stepwise association with lymph node metastasis (Fisher’s exact test, *p* < 0.001). Tumors with a score of 0 showed the lowest frequency of nodal involvement, whereas score 2 tumors demonstrated the highest frequency of lymph node metastasis. The distribution of patients across the three composite score categories is shown in Figure 3A.

This gradient became more pronounced when quantitative nodal dissemination was evaluated. Metastatic lymph node burden increased progressively across composite score categories, with median positive lymph node counts rising from 0 in score 0, to 3.5 in score 1, and 9 in score 2 tumors (Kruskal–Wallis, *p* < 0.001; Figure 3B).

A similar stepwise increase was observed for LNR, which also increased significantly with higher composite invasion scores (Kruskal–Wallis, *p* < 0.001). These findings demonstrate a consistent association between increasing composite invasion scores and greater regional nodal metastatic burden.

### 3.5. Predictive Performance of the Composite Invasion Score

**Figure 4:**
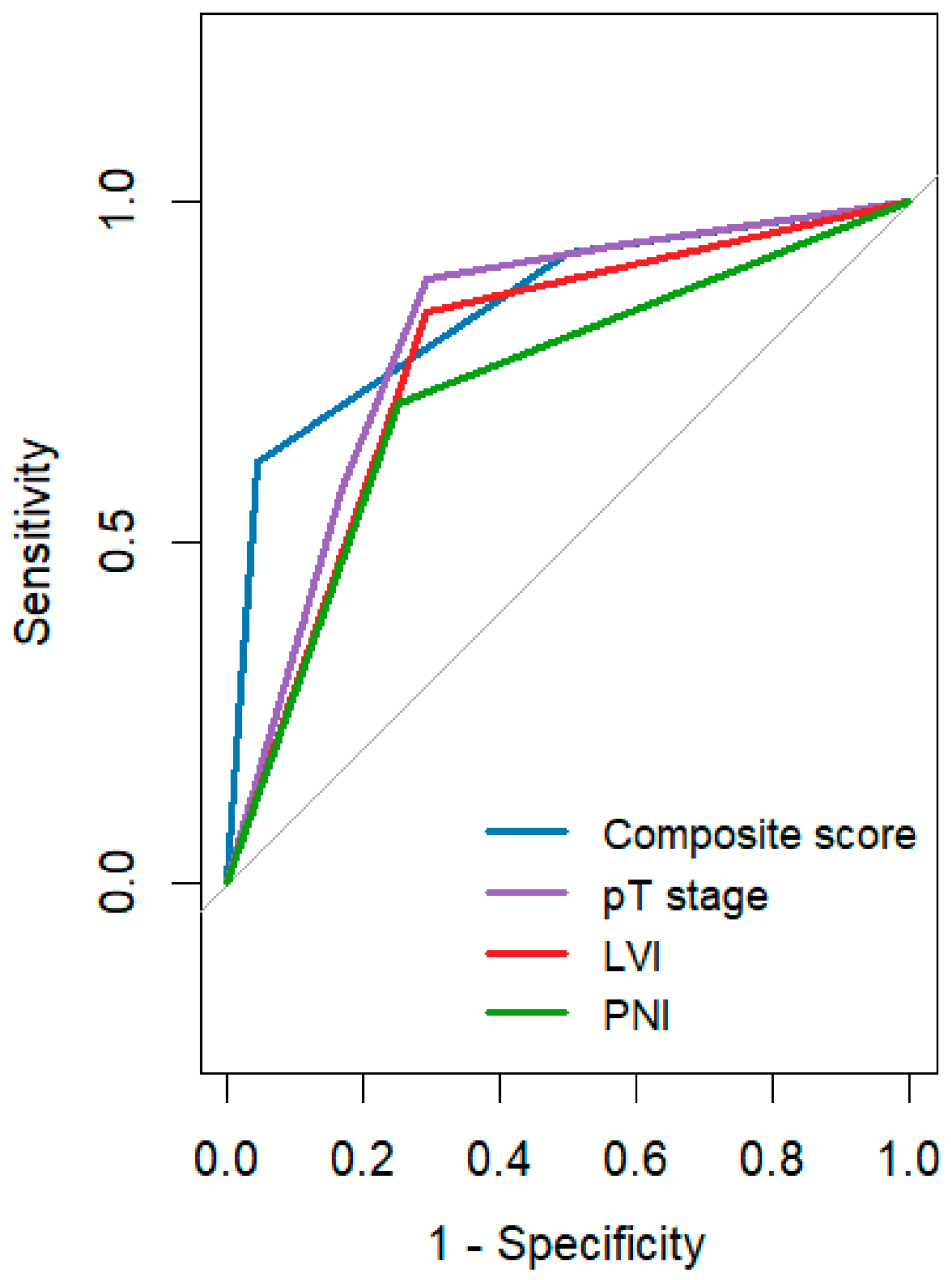
Receiver operating characteristic (ROC) curves comparing the predictive performance of the composite invasion score, pathological T stage (pT), lymphovascular invasion (LVI), and perineural invasion (PNI) for predicting lymph node metastasis. ROC analyses were performed in the complete-case–cohort (*n* = 105). The composite invasion score demonstrated the highest discriminatory performance (AUC = 0.848, 95% CI: 0.773–0.923), followed by pT stage (AUC = 0.810, 95% CI: 0.703–0.918), LVI (AUC = 0.774), and PNI (AUC = 0.727). The optimal cutoff value for the composite invasion score was 1.5, corresponding to a sensitivity of 61.7% and a specificity of 95.8%. Pairwise comparisons using DeLong’s test showed that the composite score significantly outperformed PNI (*p* = 0.002), whereas no significant differences were observed compared with LVI (*p* = 0.066) or pT stage (*p* = 0.431).

To evaluate the discriminatory performance of the composite invasion score for predicting lymph node metastasis, ROC analysis was performed in the same complete-case–cohort of 105 patients used for the multivariable logistic regression analysis. The composite invasion score demonstrated an area under the curve (AUC) of 0.848 (95% CI: 0.773–0.923), indicating good discrimination for lymph node metastasis (Figure 4). The optimal cutoff value was 1.5, corresponding to a sensitivity of 61.7% and a specificity of 95.8%.

ROC analyses were also performed for pathological T stage, lymphovascular invasion (LVI), and perineural invasion (PNI). Pairwise comparisons using DeLong’s test demonstrated that the composite invasion score significantly outperformed PNI (*p* = 0.002), whereas no statistically significant differences were observed compared with LVI (*p* = 0.066) or pathological T stage (*p* = 0.431).

These findings demonstrate that the composite invasion score provides robust postoperative discrimination for lymph node metastasis and performs at least comparably to established pathological predictors.

### 3.6. Bioinformatic Analysis of Invasion- and ECM-Related Gene Expression

To provide external molecular context for the clinicopathological findings, differential gene expression analysis was performed between early-stage (stage I–II, *n* = 59) and advanced-stage (stage III–IV, *n* = 133) gastric cancer samples using the GSE15459 dataset. Among the examined invasion- and ECM-related genes, *MMP11*, *MMP14*, and *FAP* were significantly upregulated in advanced-stage tumors compared with early-stage cases (adj.P.Val < 0.05), as presented in Table 5. *MMP11* showed the largest increase in expression (logFC = 1.34), whereas *FAP* demonstrated the strongest statistical significance (adj.P.Val = 0.008), consistent with enhanced extracellular matrix remodeling and stromal activation in advanced-stage disease. *MMP14* was also significantly upregulated, supporting increased pericellular matrix remodeling.

In addition, several ECM- and invasion-associated genes, including *VCAN*, *PDPN*, *LOX*, *PLOD2*, *P4HA1*, and *SERPINE1*, showed higher expression in advanced-stage tumors; however, these differences did not remain statistically significant after multiple testing correction.

Conversely, classical structural ECM genes (*COL1A1*, *COL1A2*, *COL3A1*, and *COL11A1*) and EMT-associated transcription factors (*TWIST1* and *PRRX1*) did not demonstrate statistically significant differential expression after adjustment for multiple comparisons.

Overall, this exploratory transcriptomic analysis demonstrates selective upregulation of genes involved in extracellular matrix remodeling and stromal activation in advanced-stage gastric cancer. Because GSE15459 does not contain annotations for lymphovascular invasion, perineural invasion, lymph node metastasis, or composite invasion score categories, these findings should be interpreted as biological context for tumor progression rather than direct transcriptomic validation of the proposed pathological scoring system.

## 4. Discussion

In the present study, invasive histopathologic features were strongly associated with regional nodal dissemination in gastric adenocarcinoma. Both LVI and PNI were significantly associated with lymph node metastasis, and each was accompanied by a higher metastatic lymph node burden. These findings are consistent with recent evidence by Zhang et al. [2], who reported that LVI and PNI were associated with lymph node metastasis and survival outcomes in gastric cancer. Their study supports the interpretation that LVI and PNI should not be regarded as merely descriptive microscopic findings, but rather as morphologic indicators of aggressive tumor biology. It should be emphasized, however, that LVI represents an earlier histopathological manifestation of the metastatic cascade, whereas lymph node metastasis reflects a subsequent stage of the same biological process. Therefore, the strong association observed between these variables should be interpreted within this biological continuum rather than as evidence that LVI is a completely independent epidemiological risk factor for lymph node metastasis. Furthermore, after adjustment for pathological T stage, histological grade, Lauren classification, age, and tumor location, the composite invasion score remained an independent predictor of lymph node metastasis, indicating that it provides prognostic information beyond established clinicopathological variables.

The clinical relevance of LVI has also been reinforced by very recent work focusing on refined detection of LVI and its subtypes. Liu et al. [3] showed that LVI, particularly lymphatic invasion, was associated with adverse prognosis and recurrence patterns in resectable gastric cancer, suggesting that more precise evaluation of invasion routes may complement conventional TNM staging. In this context, our findings extend the existing literature by integrating LVI with PNI into a cumulative composite invasion score rather than evaluating each feature in isolation. A recent systematic review and meta-analysis further confirmed that lymphatic vessel invasion is consistently associated with lymph node metastasis and adverse clinicopathological characteristics in gastric cancer, reinforcing the biological and clinical rationale for incorporating invasion-related pathological features into postoperative risk stratification models [11].

A key finding of our study was the stepwise relationship between the composite invasion score and nodal dissemination. Tumors with accumulation of invasive features showed progressively higher nodal positivity, metastatic lymph node burden, and LNR. This suggests that the coexistence of LVI and PNI may reflect increasing metastatic competence rather than a simple additive pathology finding. The composite invasion score also demonstrated good discriminatory performance for lymph node metastasis (AUC = 0.848, 95% CI: 0.773–0.923), supporting its utility as a simple postoperative pathology-derived risk stratification tool. Importantly, the proposed composite invasion score is intended as a postoperative histopathological risk stratification tool rather than a preoperative predictor of nodal status. Because both LVI and PNI are assessed on resection specimens, the score is not intended to replace pathological nodal staging. Instead, it reflects the invasive biological phenotype of the primary tumor and may complement routine pathological assessment by identifying tumors with a more aggressive invasive phenotype. Whether the composite invasion score provides incremental value for postoperative therapeutic decision-making beyond established prognostic systems remains to be determined in future studies with longitudinal clinical follow-up. This observation is consistent with current concepts emphasizing that combining complementary pathological features often provides more robust prognostic information than evaluating individual variables separately, particularly for postoperative decision-making in gastric cancer [2,12,13]. Recent radiomics work by He et al. [14] also emphasized the clinical importance of predicting LVI and PNI preoperatively, further confirming that these invasive features are increasingly viewed as key markers of tumor aggressiveness in gastric cancer.

To provide biological context for these clinicopathological observations, we performed an exploratory external transcriptomic analysis using the GSE15459 dataset. Advanced-stage tumors showed significant upregulation of *MMP11*, *MMP14*, and *FAP*, all of which are closely linked to extracellular matrix remodeling and stromal activation. *MMP11* has previously been implicated in gastric cancer invasion, and experimental studies have demonstrated that *MMP11* knockdown inhibits proliferation and invasion of gastric adenocarcinoma cells [7]. In addition, serum *MMP11* levels have been associated with clinical outcomes in advanced gastric adenocarcinoma [15].

*MMP14* also has biologic relevance to the invasive phenotype observed in our cohort. Kasurinen et al. [8] reported that high serum *MMP14* predicted worse survival in gastric cancer, and previous studies have linked *MMP14* expression with metastasis and poor prognosis. These findings support our observation that *MMP14* upregulation in advanced-stage tumors may reflect enhanced pericellular proteolysis and matrix remodeling during metastatic progression.

The significant upregulation of *FAP* in our analysis is also consistent with the growing recognition of cancer-associated fibroblasts as key regulators of gastric cancer progression. Wen et al. [16] showed that *FAP*-positive fibroblasts promote gastric cancer progression and resistance to immune checkpoint blockade. Similarly, Liu et al. [9] reported that stromal *FAP* promotes gastric cancer progression through epithelial–mesenchymal transition via the Wnt/β-catenin pathway. More recently, Lyu et al. [10] identified *FAP* as a prognostic biomarker associated with the tumor microenvironment in stomach adenocarcinoma.

Beyond individual genes, our findings fit within a broader ECM-remodeling framework. Yuan et al. reviewed how ECM remodeling contributes to tumor progression, metastasis, angiogenesis, lymphangiogenesis, and immune escape [5]. A recent gastric cancer-focused ECM study also described collagen reorganization, cancer-associated fibroblast activation, *LOX*-family activity, and collagen crosslinking as major contributors to tumor stiffness and invasion [17]. Similarly, recent comprehensive reviews have highlighted that dynamic interactions between extracellular matrix components, cancer-associated fibroblasts, immune cells, and tumor cells are fundamental drivers of invasion, metastatic dissemination, and therapeutic resistance in gastric cancer [18]. Because GSE15459 does not include annotations for lymphovascular invasion, perineural invasion, lymph node metastasis, or composite invasion score categories, these transcriptomic findings should be interpreted as biological context for stage-associated extracellular matrix remodeling rather than direct molecular validation of the proposed pathological score.

Interestingly, several ECM- and hypoxia-associated genes, including *LOX*, *PLOD2*, and *P4HA1*, showed increased expression trends in advanced-stage tumors, although they did not remain statistically significant after multiple testing correction. These observations should therefore be interpreted cautiously. Nevertheless, the direction of change is biologically plausible, as hypoxia is known to regulate ECM deposition, remodeling, and degradation, thereby promoting invasive and metastatic behavior [19,20]. Recent evidence further suggests that remodeling of the tumor microenvironment is not only a consequence of tumor progression but also actively establishes a permissive niche for metastatic dissemination through coordinated stromal, vascular, and immune interactions [21].

Our results should also be interpreted in light of studies showing that ECM remodeling is increasingly being investigated as a metastasis-associated process in gastric cancer. A recent study identified ECM remodeling-associated proteins and immune signatures as predictive markers and potential therapeutic targets for metastatic gastric cancer [22]. Although that study used proteomics, single-cell transcriptomics, and functional assays, whereas our study used histopathology and an exploratory external transcriptomic analysis, both approaches converge on the concept that metastatic gastric cancer is strongly shaped by tumor–ECM interactions. Furthermore, recent reviews of gastric adenocarcinoma emphasize that future postoperative risk assessment should integrate conventional histopathological findings with molecular and tumor microenvironment-related information to achieve more precise prognostic stratification, which is conceptually aligned with the rationale of the present composite invasion score [3,23,24].

### Study Limitations and Future Directions

This study has several limitations. First, its retrospective single-center design may limit the generalizability of the findings. Second, analyses involving lymph node metastasis, metastatic lymph node burden, lymph node ratio, and the composite invasion score were restricted to the resection cohort, and only cases with available assessments for both LVI and PNI were included in the composite score analysis. Third, information on neoadjuvant treatment was available for only a subset of patients. Consequently, the potential influence of neoadjuvant therapy on pathological T stage, lymphovascular invasion, perineural invasion, and lymph node status could not be reliably evaluated. Fourth, the pathology database did not contain longitudinal follow-up data; therefore, the prognostic value of the composite invasion score for overall survival, disease-free survival, recurrence, or its incremental prognostic value beyond established TNM staging systems could not be evaluated. Consequently, the present study should be interpreted as a clinicopathological investigation describing associations between invasive pathological features and nodal dissemination, rather than as a prognostic validation study. In addition, although the exploratory external transcriptomic analysis provided biological context for the observed clinicopathological associations, the GSE15459 dataset does not contain annotations for LVI, PNI, lymph node metastasis, or composite invasion score categories, precluding direct molecular evaluation of the proposed score.

Future studies should validate the composite invasion score in large independent multicenter cohorts with standardized pathological assessment and long-term follow-up, determine whether it provides prognostic information beyond established TNM staging and other clinicopathological models, evaluate its association with overall and disease-free survival, assess the impact of neoadjuvant therapy on the performance of the composite invasion score, and investigate whether it can improve postoperative risk stratification and adjuvant treatment decision-making. In addition, future molecular studies using datasets containing detailed annotations for LVI, PNI, and lymph node metastasis are needed to further elucidate the biological basis of the proposed score.

## Figures and Tables

**Table 1:** Baseline characteristics of the overall study cohort (*n* = 395).

Characteristic	Overall Cohort (*n* = 395)
Age, years	
Mean ± SD	66.0 ± 13.9
Median (IQR)	67 (57–76)
Sex (*n* = 395)	
Male	283 (71.6%)
Female	112 (28.4%)
Specimen type (*n* = 395)	
Biopsy	268 (67.8%)
Resection	127 (32.2%)
Lauren classification (*n* = 136)	
Intestinal	73 (53.7%)
Diffuse	52 (38.2%)
Mixed	11 (8.1%)
Biomarker assessment	
*H. pylori* positive (*n* = 149 tested)	55 (36.9%)
HER2 positive (*n* = 395)	47 (11.9%)
PD-L1 positive (*n* = 61 tested)	25 (41.0%)
dMMR (*n* = 43 tested)	5 (11.6%)
pMMR (*n* = 43 tested)	38 (88.4%)

**Table 2:** Clinicopathological characteristics of the surgical resection cohort (*n* = 126).

Characteristic	Resection Cohort (*n* = 126)
**Age, years, median (IQR)**	64 (55–75)
**Sex (*n* = 126)**	
Male	96 (76.2%)
Female	30 (23.8%)
**Tumor location (*n* = 126)**	
Cardia	11 (8.7%)
Corpus/Fundus	31 (24.6%)
Antrum/Pylorus	45 (35.7%)
Whole stomach	2 (1.6%)
Other/Unspecified	37 (29.4%)
**Lauren classification (*n* = 113)**	
Intestinal	60 (53.1%)
Diffuse	42 (37.2%)
Mixed	11 (9.7%)
**Histological grade (*n* = 112)**	
Grade 1	17 (15.2%)
Grade 2	32 (28.6%)
Grade 3	63 (56.2%)
**Neoadjuvant treatment (*n* = 62)**	
No	39 (62.9%)
Yes	23 (37.1%)
**Pathological T stage (*n* = 124)**	
pT0	10 (8.1%)
pT1	17 (13.7%)
pT2	12 (9.7%)
pT3	28 (22.6%)
pT4	57 (46.0%)
**Lymph node status (*n* = 120)**	
Negative	35 (29.2%)
Positive	85 (70.8%)
**Examined lymph nodes, median (IQR) (*n* = 121)**	34 (22–45)
**Metastatic lymph nodes, median (IQR) (*n* = 120)**	4 (0–9)
**Lymphovascular invasion (*n* = 112)**	
Absent	32 (28.6%)
Present	80 (71.4%)
**Perineural invasion (*n* = 112)**	
Absent	44 (39.3%)
Present	68 (60.7%)
**Composite invasion score (*n* = 112)**	
0	19 (17.0%)
1	38 (33.9%)
2	55 (49.1%)

**Table 3:** Association of lymphovascular invasion and perineural invasion with the number of metastatic lymph nodes in resected gastric adenocarcinoma specimens.

Feature	Absent (*n*), Median Positive Lymph Nodes (IQR)	Present (*n*), Median Positive Lymph Nodes (IQR)	*p* Value
Lymphovascular invasion (LVI)	32, 0 (0–4)	80, 6 (3–11.8)	1.038 × 10^−5^
Perineural invasion (PNI)	44, 1 (0–5)	68, 7 (3.3–14)	2.082 × 10^−6^

Data are presented as median (interquartile range, IQR). *p* values were calculated using the Wilcoxon rank-sum test.

**Table 4:** Comparative analyses of histologic subtype and biomarker status according to lymph node positivity.

Variable	Available Cases, *n*	Test	*p* Value
Lauren histologic subtype	113	Fisher’s exact test	0.718
*Helicobacter pylori* status	63	Fisher’s exact test	0.742
HER2 expression	81	Fisher’s exact test	1.000
PD-L1 status	14	Fisher’s exact test	1.000

**Table 5:** Differential expression of selected extracellular matrix remodeling and invasion-related genes between advanced-stage (III–IV) and early-stage (I–II) gastric cancer in the GSE15459 dataset.

Gene	logFC	*p* Value	adj.P.Val	Gene Function
*MMP11*	1.34	9.92 × 10^−6^	0.020	Matrix degradation, invasion
*MMP14*	0.66	3.17 × 10^−5^	0.035	ECM remodeling
*COL1A1*	0.65	1.50 × 10^−3^	0.225	Structural collagen
*COL1A2*	0.37	4.65 × 10^−3^	0.304	Structural collagen
*COL3A1*	0.35	5.02 × 10^−3^	0.313	Structural collagen
*COL11A1*	0.93	1.58 × 10^−2^	0.452	Tumor stroma collagen
*FN1*	0.43	7.52 × 10^−2^	0.648	Adhesion/migration
*POSTN*	0.38	0.123	0.709	Stromal activation
*SPARC*	0.39	2.30 × 10^−3^	0.246	ECM interaction
*VCAN*	0.75	3.10 × 10^−4^	0.120	ECM proteoglycan
*FAP*	1.02	1.62 × 10^−6^	0.008	CAF activation
*PDPN*	0.71	4.57 × 10^−4^	0.138	Cell motility
*VIM*	0.18	0.014	0.702	EMT marker
*TWIST1*	0.66	3.84 × 10^−3^	0.285	EMT transcription factor
*PRRX1*	0.65	4.03 × 10^−3^	0.293	EMT regulator
*LOX*	0.55	4.54 × 10^−3^	0.303	Collagen crosslinking
*PLOD2*	0.49	1.25 × 10^−3^	0.213	Collagen maturation
*P4HA1*	0.35	4.65 × 10^−3^	0.304	Hypoxia-related ECM
*TGFBI*	0.44	5.12 × 10^−3^	0.315	TGF-β signaling
*SERPINE1*	0.53	1.31 × 10^−2^	0.428	Invasion/metastasis

## Data Availability

The data that supports the findings of this study are available from the corresponding author upon reasonable request.

## Supplementary Materials

The following supporting information can be downloaded at: https://www.mdpi.com/article/10.3390/jcm15176509/s1, Supplementary File S1. Full list of differentially expressed genes obtained from the GEO2R analysis comparing advanced-stage (III–IV) and early-stage (I–II) gastric cancer samples in the GSE15459 dataset.
